# A mathematical model of exposure of non-target Lepidoptera to *Bt*-maize pollen expressing Cry1Ab within Europe

**DOI:** 10.1098/rspb.2009.2091

**Published:** 2010-01-06

**Authors:** J. N. Perry, Y. Devos, S. Arpaia, D. Bartsch, A. Gathmann, R. S. Hails, J. Kiss, K. Lheureux, B. Manachini, S. Mestdagh, G. Neemann, F. Ortego, J. Schiemann, J. B. Sweet

**Affiliations:** 1 Oaklands Barn, Lug's Lane, Broome, Norfolk NR35 2HT, UK; 2 European Food Safety Authority (EFSA), GMO Unit, Largo Natale Palli 5/A, 43121 Parma, Italy; 3 National Agency for New Technologies, Energy and Environment (ENEA), Research Centre Trisaia, 75026 Rotondella, Italy; 4 Bundesamt für Verbraucherschutz und Lebensmittelsicherheit (BVL), Federal Office of Consumer Protection and Food Safety, Mauerstrasse 39-42, 10117 Berlin, Germany; 5 Centre for Ecology and Hydrology, Mansfield Road, Oxford OX1 3SR, UK; 6 Plant Protection Institute, Szent István University, Pater K. 1, 2100 Gödöllő, Hungary; 7 Animal Biology Department, University of Palermo, Via Archirafi, 18, 90123 Palermo, Italy; 8 Büro für Landschaftsökologie und Umweltstudien, Wiesenstraße 8, 37073 Göttingen, Germany; 9 Centro de Investigaciones Biológicas (CSIC), Departamento de Biología de Plantas, Laboratorio Interacción Planta-Insecto, C/Ramiro de Maeztu 9, 28040 Madrid, Spain; 10 Julius Kühn Institute, Federal Research Centre for Cultivated Plants (JKI), Institute for Biosafety of Genetically Modified Plants, Erwin-Baur-Strasse 27, 06484 Quedlinburg, Germany; 11 Sweet Environmental Consultants, 6 The Green, Willingham, Cambridge CB24 5JA, UK

**Keywords:** genetically modified maize, Cry1Ab, non-target Lepidoptera, mathematical model, exposure, risk assessment

## Abstract

Genetically modified (GM) maize MON810 expresses a Cry1Ab insecticidal protein, derived from *Bacillus thuringiensis* (*Bt*), toxic to lepidopteran target pests such as *Ostrinia nubilalis*. An environmental risk to non-target Lepidoptera from this GM crop is exposure to harmful amounts of *Bt*-containing pollen deposited on host plants in or near MON810 fields. An 11-parameter mathematical model analysed exposure of larvae of three non-target species: the butterflies *Inachis io* (L.), *Vanessa atalanta* (L.) and moth *Plutella xylostella* (L.), in 11 representative maize cultivation regions in four European countries. A mortality–dose relationship was integrated with a dose–distance relationship to estimate mortality both within the maize MON810 crop and within the field margin at varying distances from the crop edge. Mortality estimates were adjusted to allow for physical effects; the lack of temporal coincidence between the susceptible larval stage concerned and the period over which maize MON810 pollen is shed; and seven further parameters concerned with maize agronomy and host-plant ecology. Sublethal effects were estimated and allowance made for aggregated pollen deposition. Estimated environmental impact was low: in all regions, the calculated mortality rate for worst-case scenarios was less than one individual in every 1572 for the butterflies and one in 392 for the moth.

## 1. Introduction

Several genetically modified (GM) crops have been developed to provide protection against certain lepidopteran target pests, such as the European corn borer *Ostrinia nubilalis* (Hübner) and the Mediterranean corn borer, *Sesamia nonagrioides* (Lefebvre), by the introduction of a part of a *Bacillus thuringiensis* (*Bt*) gene encoding the insecticidal Cry1Ab protein (http://www.agbios.com/dbase.php). The *Bt* protein binds to specific receptors on the epithelial surface of the midgut of lepidopteran species, leading to the death of larvae through pore formation, cell burst and septicaemia (Crickmore 2005; Soberón *et al*. 2009). At present, maize MON810 is the only commercial *Bt* crop grown in the European Union (EU), having been cropped over a significant area since 2003, mainly in Spain (Gómez-Barbero *et al*. 2008).

Within Europe, maize is not an important food resource for indigenous lepidopteran larvae, with the exception of few pest species, so exposure to potentially harmful amounts of *Bt*-containing pollen deposited on host plants in or near maize MON810 fields is the main risk to non-target Lepidoptera, as reviewed in the BEETLE report (2009). A laboratory assay suggested a hazard to the North American Monarch butterfly (*Danaus plexippus* L.) larvae that consumed maize *Bt*11 pollen deposited on milkweed (*Asclepias* spp., especially common milkweed, *Asclepias syriaca* L.) leaves compared with those reared on leaves dusted with non-GM maize pollen or on leaves without pollen (Losey *et al*. 1999). Subsequently, lethal and sublethal effects of *Bt*-maize pollen consumption by lepidopteran larvae have been reported for several non-target lepidopteran species under laboratory conditions in the USA (Jesse & Obrycki 2000; Wraight *et al*. 2000; Hellmich *et al*. 2001; Dively *et al*. 2004; Anderson *et al*. 2005; Mattila *et al*. 2005) and within Europe (Felke *et al*. 2002; Felke & Langenbruch 2005; Lang & Vojtech 2006), the magnitude of the hazard being dependent on the *Bt*-maize event, the lepidopteran species and the larval stage, the amount of pollen consumed and amount of Cry1Ab protein ingested.

The hazard reported for the Monarch butterfly in laboratory experiments (Losey *et al*. 1999) led to extensive exposure assessments in the field in the USA, which found that the proportion of Monarch butterfly populations exposed to toxic levels of *Bt*-pollen is small owing to the limited spatial distribution of maize pollen (Pleasants *et al*. 2001) and the limited temporal overlap between larval development and pollen shed (Oberhauser *et al*. 2001). Exposure to potentially harmful quantities of maize pollen is largely restricted to pollen deposited on milkweed host plants in the area of field margins within 1–5 m of the edge of maize fields, since the highest pollen concentrations occur in and near maize fields. A risk assessment model estimated that the average probability of short-duration exposure to *Bt*-maize pollen within maize fields, for those states and provinces within the corn belt that constitute 50 per cent of the eastern North American Monarch breeding habitat, was less than 0.1 per cent (Sears *et al*. 2001). Comparable conclusions were reached by a similar approach applied to the risk associated with longer term exposure of Monarch butterfly populations (Dively *et al*. 2004).

Extensive exposure assessment studies similar to those performed in the USA have not been conducted under European environmental conditions, although Darvas *et al*. (2004) and Gathmann *et al*. (2006*b*) conducted exposure and abundance studies of certain species of non-target Lepidoptera in specific localities in Europe. Information regarding the scale of the hazard is available from estimates of the number of non-target macrolepidopteran species that might theoretically be exposed and thus affected on a regional scale by *Bt*-maize pollen (Schmitz *et al*. 2003; Darvas *et al*. 2004; Traxler *et al*. 2005). In addition, other field studies provide some relevant data on the exposure of European lepidopteran species in agricultural landscapes on a population level (Lang *et al*. 2004; Gathmann *et al*. 2006*a*,*b*), but data on some aspects of exposure, particularly plant–insect phenology, pollen consumption and subsequent mortality in field conditions, are rare within Europe. Although the cultivation of *Bt*-maize has been ongoing for several years in Spain, data from Spain on effects on non-target Lepidoptera are scarce because their abundance tends to be low at the time when maize is pollinating. Extrapolating observations made on one non-target lepidopteran species to another is problematic because of between-species variability in both acute sensitivity to Cry1Ab protein and plant–insect phenological coincidence (Schmitz *et al*. 2003). Moreover, the MON810 event has now been integrated into many commercial maize varieties with differing sowing dates and developmental characteristics, resulting in a range of flowering dates, thus increasing temporal variability in exposure to the Cry1Ab protein (Van Hout *et al*. 2008). Aviron *et al*. (2009) also emphasized the practical difficulties of conducting experiments to detect small effects, where they exist, on all lepidopteran species that could be potentially exposed to *Bt*-maize pollen.

Under EU Directive 2001/18/EC on the deliberate release into the environment of GM organisms, there is a legal requirement for an environmental risk assessment of the cultivation of maize MON810 within the EU (EFSA 2009); this must include an assessment of the adverse effects on non-target Lepidoptera resulting from exposure to pollen from this *Bt*-maize (see Sears *et al*. 2001; Wolt *et al*. 2005; Peterson *et al*. 2006). This paper describes a mathematical model used to facilitate the quantification of risk assessment. The model explores possible scenarios for the exposure of three widespread European lepidopteran species in 11 representative maize cultivation regions in four European countries. For the first time, to our knowledge, the integration of a mortality–dose relationship from the laboratory with a dose–distance relationship from the field allows direct estimates of larval mortality both within the crop and within the field margin as a function of distance from the crop edge. These estimates were adjusted to allow for physical effects; the lack of temporal coincidence between the susceptible larval stage concerned and the period over which maize MON810 pollen is shed; and seven further exposure parameters concerned with maize agronomy and host-plant ecology. Sublethal effects were estimated and allowance made for aggregated pollen deposition. At each stage in the model development, where there was a choice we have endeavoured to model ‘worst-case’ scenarios, in which any assumptions would tend towards overestimation rather than underestimation of mortality and sublethality.

## 2. Derivation of model

### (a) Parameters

The model has 11 parameters. The principal parameters are denoted *g* and *h*. The parameter *g*(*E*) represents the worst-case probability that a given larva will suffer mortality from ingesting maize MON810 pollen deposited onto its host plant located in the field margin at distance *E* from the nearest edge of the maize MON810 crop. Here, the term worst-case refers to potential mortality, as measured in the laboratory or under controlled experimental conditions, before allowance for factors such as physical effects and temporal coincidence, which reduce this mortality to realistic values observed in the field (see below). The parameter *g* represents an average probability over factors such as whether the margin is on the upwind or downwind side of the field, or time of day. The parameter *h* represents the worst-case probability that a given larva will suffer mortality from ingesting maize MON810 pollen deposited onto its host plant located within the maize MON810 crop.

Two parameters model effects that reduce exposure. The parameter *x* represents the proportion of larvae that remains exposed, after allowance for a set of physical effects that include: degradation of pollen toxicity (Griego & Spence 1978; Obrist *et al*. 2006); rain washing pollen off leaves (Pleasants *et al*. 2001); larvae feeding on the underside of leaves where pollen densities are smaller (Jesse & Obrycki 2003); larval avoidance of leaf midrib area where pollen densities tend to be aggregated (Pleasants *et al*. 2001); larvae feeding on lower leaves on which less pollen has been deposited through the shading effect of leaves above them (Pleasants *et al*. 2001), etc. The parameter *a* represents the proportion by which exposure is reduced owing to lack of temporal coincidence between the susceptible larval stage concerned and the period over which maize MON810 pollen is shed (Wolt *et al*. 2003; Lang *et al*. 2004; Gathmann *et al*. 2006*b*). For simplicity, this model considers a single larval instar. The quantification of temporal coincidence is conceptualized as follows. Lepidopteran larvae do not enter instars synchronously; it is assumed that there is a bivariate distribution for the days on which larvae enter (*t*_s_) and leave (*t*_l_) the particular susceptible instar modelled. Regarding anthesis, similarly, the period within the flowering of maize when pollen is shed is asynchronous between individual crop plants, and it is assumed that there is a bivariate distribution for the days on which plants begin (*t*_b_) and end (*t*_e_) shedding pollen. Consider the ellipsoids that represent the 95th percentiles of these two bivariate distributions. This model assumes that the full exposure of larvae to pollen is reduced proportionately by the degree to which the overlap between these two ellipsoids is incomplete. Specifically, the parameter *a* measures the proportion of the ellipsoid that represents the 95th percentile for instar development that is not overlapped by the ellipsoid that represents the 95th percentile for pollen shed. Note that the expected actual mortality rate for an individual larva is therefore *xag*(*E*) within the field margin and *xah* within the crop.

The parameters *g*(*E*) and *h* are specific to the host plant, lepidopteran species and larval stage modelled, but generic across regions; *x* and *a* are assumed to be specific to the geographical region. The following three parameters of the model control large-scale demographic aspects of exposure and are all specific to regions but generic across host plant and species. The parameter *z* represents the proportion of arable fields that are cropped with maize in any given year in the defined region. The parameter *v* represents the proportion of maize sown within the defined region, that is, the variety MON810 (Gómez-Barbero *et al*. 2008). The parameter *y* represents the proportion of the lepidopteran host plant that is found within arable crops and in their margins (as opposed to gardens, woodlands, non-arable fields, etc., which are too far from maize MON810 fields for pollen deposition to present any quantifiable risk). Note that the proportion of the population potentially exposed, after allowance for reduction owing to these large-scale demographic factors, is then *yzv*. The next two parameters are specific to regions, host plants and species; parameters *e* and *f* measure, respectively, the density of the host plant within the maize crop and in the field margins, in plants m^−2^. For simplicity, it is assumed that host plants occur spatially at random within crops and field margins. The final two parameters relate to field size and are therefore specific to regions but generic across host plants and species. For simplicity, it is assumed that maize MON810 fields are square. The parameter *C* represents the average size of a maize field in hectares and the parameter *D* represents the average width of a field margin in metres. When parametrizing the model for specific regions, allowance is made for possible bimodal distributions of field margins because fields in a region may often have margins that are several metres wide or have no margins at all.

### (b) Species and regions

The model was parametrized for exposed larvae of three non-target lepidopteran species: the peacock butterfly *Inachis io* L. (Lepidoptera Nymphalidae), the red admiral butterfly *Vanessa atalanta* L. (Lepidoptera Nymphalidae) and the diamondback moth *Plutella xylostella* L. (Lepidoptera Plutellidae), for 11 representative European maize cultivation regions: Aachen, Berkatal, Bonn, Grebbin, Oderbruch and the Upper Rhine Valley from Germany; the Po Valley (east and central) and the Po Valley (southern and coastal) in Italy; Tolna County in Hungary; and the Ebro Valley and Madrid from Spain. For both butterflies, the model was parametrized for the most susceptible first instars (Darvas *et al*. 2004), and the widespread nettle host plant *Urtica dioica* L. For the moth, the model was parametrized for fourth instar larvae, which were the most sensitive of the species and instars studied by Felke & Langenbruch (2005), and which were assumed to feed on widespread Brassicaceae host plants such as *Lepidium draba* (L.), *Capsella bursa-pastoris* (L. Medik.), *Thlaspi arvense* (L.) and *Raphanus raphanistrum* (L.). The two butterfly species were chosen for their conservation value (Schmitz *et al*. 2003; Darvas *et al*. 2004; Traxler *et al*. 2005), while the moth, a serious pest of cultivated Cruciferae, was chosen because it is one of the most susceptible Lepidoptera to the Cry1Ab insecticidal protein (Felke & Langenbruch 2005). In controlled conditions, unprotected larvae of *V. atalanta* are thought to be of very similar susceptibility to those of *I. io* (Darvas *et al*. 2004). Both species are somewhat protected under field conditions from pollen deposition; the former species creates ‘leaf bags’, the latter builds webs (e.g. Scott 1986).

### (c) Estimates of individual mortality

In this section: (i) published mortality–dose relationships, for individuals of the three lepidopteran species with pollen of the different event maize *Bt*176, are calibrated to relate to maize MON810 pollen and expressed in terms of logits; (ii) published pollen deposition–distance relationships for pollen deposited on the different host plant *A. syriaca* are calibrated to relate to *U. dioica* and Brassicaceae; and (iii) results from (i) and (ii) are integrated to yield field margin and within-crop mortality–distance relationships for individual larvae of the chosen larval/host-plant combinations.

In no-choice feeding studies with leaf discs coated with maize *Bt*176 pollen, Felke & Langenbruch (2005) showed that the average lethal concentration value that kills half (LC_50_) of the fourth instars of *P. xylostella* was eight pollen grains placed on a 0.071 cm^2^ host-plant leaf disc (113.1 pollen grains cm^−2^ leaf area). Results for other lepidopteran larvae included *O. nubilalis*, LC_50_ 452.6 pollen grains cm^−2^ leaf area for second instars, and *I. io* (see especially Felke *et al*. in press), LC_50_ 186.8 pollen grains cm^−2^ leaf area for first instars. Since concentrations of the Cry1Ab protein in pollen of maize MON810 are approximately 31-fold less than those in maize *Bt*176 (Hellmich *et al*. 2001; Sears *et al*. 2001; Mendelsohn *et al*. 2003; Nguyen & Jehle 2007), the LC_50_ for MON810 was estimated as 3626 pollen grains cm^−2^ leaf area for fourth instars of *P. xylostella* and 5800 pollen grains cm^−2^ leaf area for first instars of *I. io* and *V. atalanta*.

Quantification of the mortality–dose relationship through a probit- or logit-regression relationship requires information concerning not only the intercept (effectively given by the LC_50_) but also the slope (the rate of change of mortality with change in concentration). Estimates of slopes are scarce for non-target Lepidoptera, but Felke *et al.* (in press) estimated the probit slope to be in excess of 5.0. Considering that pollen concentrations on host plants through field deposition are likely to be considerably smaller than the LC_50_ value (Wraight *et al*. 2000), such a large slope implies very low mortality rates at concentrations typically encountered in or near fields. Indeed, for the slope estimate given by Felke *et al*. (in press), the value of the parameter *h* for first instars of *I. io* would be less than 10^−9^, and the value of rate g(*E*) within field margins would be even smaller. Whether such estimates are realistic in the field is yet to be determined. However, as a worst-case scenario, the model was parametrized with a much smaller estimate of the probit slope, 1.095, an estimate obtained by Saeglitz *et al*. (2006*a*,*b*) for *O. nubilalis* over a number of experiments. This compares reasonably well with estimates made by Farinós *et al*. (2004), which for *O. nubilalis* were in the range 1.33–3.15, and for *S. nonagrioides* were in the range 0.92–2.96. The value 1.095 was used throughout for all three species considered. Since the slope is invariant under the change of scale for probit analysis, the estimated slope applies equally both to concentrations expressed in units of pollen grains per square centimetre leaf area and to doses in units of nanogram of truncated toxin used by Saeglitz *et al*. (2006*a*,*b*).

Hence, if *p*_B_ represents the proportion of *I. io* and *V. atalanta* individuals that suffer mortality as a result of a concentration, *d*, expressed as the number of maize MON810 pollen grains cm^−2^, then
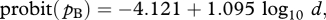

and if *p*_M_ represents the proportion of the *P. xylostella* individuals that suffer mortality, then
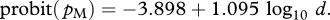



Working with logits rather than probits yields an exact solution for expressions involving *p.* The above probit-regression relationships are well approximated by the following logit-regression relationships:
2.1
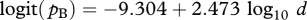

and
2.2
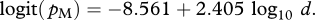



The concentration, *d*, of pollen grains adhering to the leaves of host plants declines rapidly with increasing distance in metres, *E*, from the maize source (Eastham & Sweet 2002; Jarosz *et al*. 2004; Devos *et al*. 2005). This decline in pollen deposition within the margins of maize fields was modelled through a linear regression of log_10_ *d* on *E*, using the extensive data of Wraight *et al*. (2000, especially fig. 2). Wraight *et al*. (2000) measured maize pollen falling on microscope slides covered with a coat of petroleum jelly. The slides provided an accurate measure of total pollen deposition but probably overestimated the amount of pollen retained on foliage by about threefold (Pleasants *et al*. 2001; Lang *et al*. 2004). Allowance for this results in the following relationship:
2.3
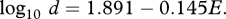



This relationship is consistent with results of Jesse & Obrycki (2000) from a smaller set of data, and is appropriate for *P. xylostella* larvae feeding on Brassicaceae. However, for larvae of *I. io* and *V. atalanta*, the results of Darvas *et al*. (2004) suggest that about 2.85 times as much pollen adheres to the hairy leaves of its host plant *U. dioica*, suggesting that the relationship be amended to
2.4
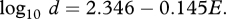



For larvae of *I. io* and *V. atalanta*, equations (2.1) and (2.4) may be combined to express mortality within the field margin directly in terms of distance from the edge of the crop, thus yielding the parameter denoted *g*(*E*) above:
2.5
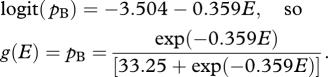



Note that at the very edge of a maize MON810 crop, where *E* = 0, the estimated mortality rate is *g*(0) = *p*_B_ = 0.0292 (equivalent to one individual in 34.25), and that 2 m into the margin, this rate itself is approximately halved. Numerical integration shows that average mortality within a 2 m band within the margin is 0.0209 (cf. with the value of 0.075 for the corresponding parameter conjectured by the EFSA GMO Panel; EFSA 2009). For larvae of *P. xylostella*, equations (2.2) and (2.3) may be combined similarly to yield
2.6
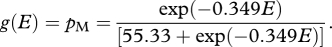



Estimates of the parameter *h* may be derived by noting that pollen deposition within a maize crop is approximately 2.7 times that at the edge (Jesse & Obrycki 2000; Wraight *et al*. 2000; Pleasants *et al*. 2001). For larvae of *I. io* and *V. atalanta*, the adjusted equations (2.1) and (2.4) are combined to give
2.7
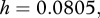

and for larvae of *P. xylostella* a value of
2.8
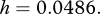



**Figure 1.**
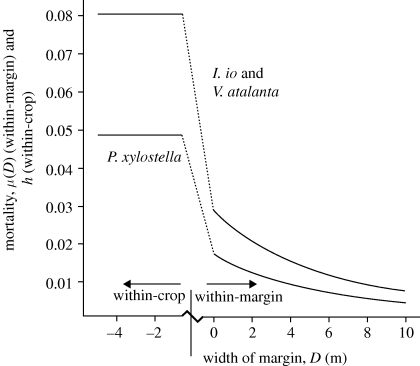
Estimated values of the parameter values *h* (worst-case within-crop mortality, assumed constant over the crop) and *μ*(*D*) (worst-case average mortality within a field margin of width *D* metres, declining with *D*) for the butterflies *I. io* and *V. atalanta* and the moth *P. xylostella*. Here, the term worst-case refers to potential mortality, as measured in the laboratory or under controlled conditions, before allowance for factors such as physical effects and temporal coincidence, and as opposed to more realistic values observed in the field (see text for all factors considered).

The expected proportion of all the larvae in a margin that suffer worst-case mortality, before allowing for effects that reduce exposure, is denoted by *μ* = *μ*(*D*), and is obtained by averaging the value of *g*(*E*) over the margin. In practice, *μ* is obtained by the numerical integration of equation (2.5) or (2.6), between the values *E* = 0 and *E* = *D*. Values of *μ* are shown for various margin widths, for the butterflies and the moth, in figure 1. For all species considered, the parameter *μ*(*D*) is about double the value for small values of *D* (very narrow margins) than for *D* = 5, and there is a further approximate halving of *μ*(*D*) at *D* = 10. Estimates of *ax* are typically about one-third, so the estimated actual within-crop mortality of individual larvae is about 2–3% for *I. io* and *V. atalanta* and about 1–2% for *P. xylostella,* and the estimated actual within-margin mortality of individual larvae, for any width of margin, is always less than 1 per cent for *I. io* and *V. atalanta* and less than 0.6 per cent for *P. xylostella*.

### (d) Estimates of population mortality

For an assumed specific square field of size *C* (ha) with a margin of *D* (m), the within-margin area is approximately 400*D*√*C* (m^2^), so the expected number of host plants within the field margins is 400*Df*√*C*. The expected number of host plants within the crop is 10 000*Ce*. Then, of the population that is potentially exposed within the crop and field margins, an approximate proportion (10 000*Ceh +* 400*fD*√C*μ*)/(10 000*Ce +* 400*fD*√*C*) suffers mortality, which may be simplified to (25*eh*√*C* + *fD*μ**)/(25*e*√*C* + *fD*). After allowance for large-scale demographic effects, physical effects, temporal overlap and spatial overlap, the estimated proportion of the population that suffers mortality is2.8
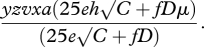



For regions where margins have a bimodal distribution for which there is a probability of *q* that a field will have no margin at all, then this proportion becomes
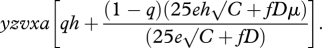



Values of parameters other than *g*, *μ*, and *h* were chosen by the authors separately for each region modelled. Information on the parameter *v* for maize MON810 is only available from the larger *Bt-*maize cultivation areas in Spain, where it has reached 0.65 in some regions. Hence, for the regions modelled in Germany, Hungary and Italy, the maximum limit for *Bt-*maize (*v* = 0.8) was chosen (based on current insect resistance management requirements for lepidopteran target pests, requiring 20% non-*Bt-*maize refuge areas), to ensure that a worst-case scenario was modelled that would yield relatively large estimates of mortality. A summary of all chosen parameter values is given in table 1.

**Table 1.** Parameter values used in the model. (Values given within curly brackets {,,,} are, respectively, the number of regions considered, and the minimum, maximum and median of authors' estimates across regions. For parameter *v*, values for nine of 11 regions (all those outside Spain) were set at 0.8, to model the worst-case scenario for potential uptake, defined as that which gives maximal predictions of mortality.)

parameter	type (units)	species *I. io* on *U. dioica*	*V. atalanta* on *U. dioica*	*P. xylostella* on Brassicaceae	derivation
margin mortality, *g*(*E*)	probability (—)	equation (2.5)	equation (2.5)	equation (2.6)	calculated
within-crop mortality, *h*	probability (—)	0.0805	0.0805	0.148	calculated
physical effects, *x*^a^	proportion (—)	{10, 0.2, 0.8, 0.4}	{10, 0.1, 0.7, 0.4}	{11, 0.1, 0.8, 0.5}	
temporal coincidence, *a*^a^	proportion (—)	{10, 0.01, 0.6, 0.5}	{10, 0.01, 0.6, 0.5}	{11, 0.1, 0.8, 0.5}	
maize cropping, *z*^a^	proportion (—)		{11, 0.024, 0.7, 0.2}		generic across species
utilization rate, *v*^a^	proportion (—)		{11, 0.035, 0.8, 0.8}		set as worst-case outside Spain, and generic across species
host plant in arable, *y*^a^	proportion (—)	{11, 0.1, 0.5, 0.23}	{11, 0.1, 0.5, 0.23}	{11, 0.1, 0.8, 0.5}	
host plant within-crop, *e*^a^	density (m^−2^)	{11, 0, 0.01, 0}	{11, 0, 0.01, 0}	{11, 0, 0.5, 0}	
host plant in margin, *f*^a^	density (m^−2^)	{11, 0, 10.0, 0.5}	{11, 0, 10.0, 0.5}	{11, 0, 15.0, 0.5}	
size of maize fields, *C*^a^	area (ha)		{11, 1.1, 58.0, 15.0}		generic across species
width of margin, *D*^a^	distance (m)		{11, 1.0, 4.5, 2.0}		generic across species

^a^All parameters vary regionally and were estimated by authors.

### (e) Aggregated pollen deposition

While it is generally agreed that densities of pollen grains decline rapidly with increasing distance from the maize source (Eastham & Sweet 2002; Jarosz *et al*. 2004; Devos *et al*. 2005), few studies have measured the variability of pollen concentrations. Pollen deposition may vary greatly spatially depending upon weather conditions (Jarosz *et al*. 2004; Hofmann 2009). Owing to vertical wind movements or gusts, particularly in thundery conditions on summer afternoons, a particular small area may experience a larger than average concentration of pollen, even many tens of metres away from a maize field (Vogler *et al*. 2009). However, such larger than average pollen concentrations are balanced by smaller than average values elsewhere, where effects are diluted. The frequency distributions given in table 2 of Pleasants *et al*. (2001) demonstrate that this variability may be considerable. This effect has been quantified in fig. 4 of Hofmann (2007), where log_10_ *d* is shown to decline linearly with log_10_ *E*, as expected, but with variability about the regression line that may be represented by a normal random variable with variance about 0.175, so that the 99% confidence interval around the line encompasses about 12-fold variation in concentration, at any given distance, in both directions. For linear systems, the average expected effect of such variation would be zero, but the model considered here is highly nonlinear; therefore, it is necessary to assess what is the effect of the measured variability on the deterministic estimates of mortality.

This was done by simulating, for each of a range of distances *E*, a thousand random variables *N*, where *N* is a normal random variable with zero mean and variance 0.175, and computing log_10_ *d* = 1.891 – 0.145*E* + *N*, the stochastic analogue of equation (2.4) and, in separate simulations, log_10_ *d* = 2.346 – 0.145*E*, the stochastic analogue of equation (2.6). From that set of values, equations (2.1) and (2.2) were used to derive estimates of mortality that allowed for stochastic variability in pollen deposition, for comparison with predictions from the deterministic case derived in equations (2.5) and (2.6).

### (f) Sublethal effects

There is little data concerning sublethal effects available to parametrize models, so our methods are subject to considerable uncertainty. However, if sublethality (Dively *et al*. 2004; Lang & Vojtech 2006) is defined as a reduction in larval weight gain, then observations in Spain on the lepidopteran pests *O. nubilalis* and *S. nonagrioides* indicate that mortality rates caused by maize MON810 pollen of about 25 and 10 per cent correspond to sublethality rates of about 100 and 50 per cent, respectively. These values are broadly consistent with the data of Lang & Vojtech (2006) and Felke *et al*. (2002). Therefore, sublethal effects were modelled by assuming that, for any given width of margin *D*, and the average worst-case mortality rate within the margin of *μ*(*D*), the proportion of larvae suffering sublethal effects, before allowance for any effects that reduced exposure, was 4*μ*(*D*). Similarly, within the crop, worst-case sublethality rates before allowance for other effects were assumed to be four times worst-case mortality rates, i.e. 0.322 for larvae of *I. io/V. atalanta* and 0.194 for larvae of *P. xylostella*. A key effect of Cry toxins on lepidopteran larvae is reduced feeding, which can lead to a greatly reduced rate of development and a level of functional mortality much greater than estimates of direct mortality measured in laboratory assays over short periods. Under a worst-case scenario, all larvae suffering sublethal effects would be assumed to die without completing their development, although this would most probably greatly overestimate the actual mortality.

## 3. Results

Estimates of population mortality and sublethality rates are given for all species and regions considered in table 2. For the two butterflies, estimates for Spain were zero because for those regions, at the time of maize pollination, there are almost no host plants for larvae to feed on in the fields or field margins, since weed control and local irrigation culture suppress weed and field margin vegetation strongly. Indeed, intensive field surveys in 2009 recorded no *U. dioica* plants and only one species, *C. draba* (L.), of Cruciferae, with no lepidopteran larvae feeding on this weed (G. P. Farinós 2009, personal communication). For each species, the minimum, maximum and median rates, excluding Spain, were computed over the regions considered. The mean of the stochastic analogues of the parameter *g*(*E*), calculated using the variability described in Hofmann (2007), was, for *I. io* and *V. atalanta*, between 1.53 (for *E* = 0) and 1.63 (for *E* = 4.5) times greater than the deterministic value shown in figure 1. For *P. xylostella*, the mean of the stochastic analogue was between 1.71 (for *E* = 0) and 1.79 (for *E* = 4.5) times greater than the deterministic value. After allowance for all the effects modelled, the estimated median stochastic mortality rate over regions (excluding Spain) for both *I. io* and *V. atalanta* was 2.66 × 10^−4^ (1.33 times the corresponding deterministic value), and for *P. xylostella* was 2.67 × 10^−4^ (1.17 times the corresponding deterministic value).

**Table 2.** Estimated population mortality rates. (For the butterflies, *I. io* and *V. atalanta*, the computed minimum, maximum and median values exclude Spain.)

	mortality			sublethality		
region	*I. io*	*V. atalanta*	*P. xylostella*	*I. io*	*V. atalanta*	*P. xylostella*
Bonn	2.95 × 10^−5^	2.95 × 10^−5^	6.11 × 10^−5^	1.18 × 10^−4^	1.18 × 10^−4^	2.44 × 10^−4^
Oderbruch	5.03 × 10^−5^	5.03 × 10^−5^	6.16 × 10^−5^	2.01 × 10^−4^	2.01 × 10^−4^	2.46 × 10^−4^
Aachen	1.68 × 10^−4^	1.68 × 10^−4^	6.16 × 10^−6^	6.70 × 10^−4^	6.70 × 10^−4^	2.46 × 10^−5^
Berkatal	2.32 × 10^−4^	2.32 × 10^−4^	3.04 × 10^−4^	9.29 × 10^−4^	9.29 × 10^−4^	1.22 × 10^−3^
Grebbin	6.36 × 10^−4^	6.36 × 10^−4^	7.69 × 10^−4^	2.55 × 10^−3^	2.55 × 10^−3^	3.08 × 10^−3^
Upper Rhine Valley	4.40 × 10^−4^	4.40 × 10^−4^	2.55 × 10^−3^	1.76 × 10^−3^	1.76 × 10^−3^	1.02 × 10^−2^
Tolna County	1.91 × 10^−5^	9.57 × 10^−6^	1.53 × 10^−4^	7.65 × 10^−5^	3.83 × 10^−5^	6.11 × 10^−4^
Po Valley (central)	4.06 × 10^−4^	3.55 × 10^−4^	9.79 × 10^−4^	1.62 × 10^−3^	1.42 × 10^−3^	3.92 × 10^−3^
Po Valley (coastal)	—	—	5.13 × 10^−5^	—	—	2.05 × 10^−4^
Madrid	0	0	1.00 × 10^−9^	0	0	3.00 × 10^−9^
Ebro Valley	0	0	2.30 × 10^−8^	0	0	9.00 × 10^−8^
minimum over regions	1.91 × 10^−5^	9.57 × 10^−6^	1.00 × 10^−9^	7.65 × 10^−5^	3.83 × 10^−5^	3.00 × 10^−9^
maximum over regions	6.36 × 10^−4^	6.36 × 10^−4^	2.55 × 10^−3^	2.55 × 10^−3^	2.55 × 10^−3^	1.02 × 10^−2^
median over regions	2.00 × 10^−4^	2.00 × 10^−4^	2.29 × 10^−4^	8.00 × 10^−4^	8.00 × 10^−4^	9.14 × 10^−4^

## 4. Discussion

The conclusions from this modelling exercise are that a full exposure assessment is possible for several non-target lepidopteran species exposed to Cry1Ab expressing pollen in or near maize MON810 fields, but it requires many factors to be taken into account, some of which have had to be modelled with restricted available data. However, we believe that the predictions made here are relatively robust for the following reasons. First, the estimates for larvae of non-target Lepidoptera reported here represent worst-case scenarios, in which any assumptions would tend towards overestimation, rather than underestimation of mortality. The estimates were probably most sensitive to the variable measuring the rate of change of mortality with concentration, i.e. the slope in the probit analysis. Indeed, had the estimate of Felke *et al*. (in press) been used instead of that of Saeglitz *et al*. (2006*a*,*b*) the mortality rates reported would have been about 8 × 10^−7^ smaller. Furthermore, while future utilization rates measured by parameter *v*, cannot now be known accurately, the assumed rate of 0.8 is most unlikely to be achieved even if cultivation were unrestricted. Therefore, population mortality rates would most probably be even smaller than reported here. Second, the stochastic simulations suggested that the increase in mortality rates owing to aggregation of pollen deposition into heterogeneous clumps will be no more than one-third of those reported in table 2. Third, for *P. xylostella*, while there can be marked variance in the sensitivity of different unselected populations to Cry toxins and while the species, cultivar and age of the leaf of the brassica used can also significantly influence the toxicity in bioassays, the sensitivity of the population used by Felke & Langenbruch (2005), from which our data derives, was relatively high. Fourth, the model used here suggests that population sublethality rates would be likely to be proportionally larger than population mortality rates by the same multiplicative factor that sublethality was assumed to exceed mortality for individual larvae. In that case, future estimates of population sublethality might usefully be based on estimates for individuals from laboratory studies.

Predicted environmental impact on the studied non-target lepidopteran larvae owing to exposure to potentially harmful amounts of pollen deposited on host plants in or near maize MON810 fields was low. In all regions: Aachen, Berkatal, Bonn, Grebbin, Oderbruch and the Upper Rhine Valley from Germany; the Po Valley (central) and the Po Valley (coastal) from Italy; Tolna County in Hungary; and the Ebro Valley and Madrid from Spain, the calculated mortality rate was less than 6.36 × 10^−4^ (one individual in every 1572) for the butterflies *I. io* and *V. atalanta* and 2.55 × 10^−3^ (one individual in every 392) for the *moth P. xylostella*. The median (typical) rates across all regions excluding Spain were 2 × 10^−4^ (one individual in every 5000) for the butterflies and 2.29 × 10^−4^ (one individual in every 4366) for the moth.

Our results suggest that previous estimates (EFSA 2009), using similar techniques but relying on experts' estimates for parameters *g*(*E*) and *h* rather than calculated values, were overly cautious and that mortality and sublethality are about four times less than they estimated.

To place the above results into a quantified population-dynamic context, it would be necessary to predict the precise effects of mortality owing to *Bt*-maize MON810 in a particular generation(s) on succeeding generations. This would require the accurate determination of key factors from life table data (Varley *et al*. 1973), which is beyond the scope of this study. However, some rough indication may be given by noting that the greatest mortality predicted was 0.00255 for *P. xylostella* in the Upper Rhine Valley. The number of generations per year of *P. xylostella* might be up to three in Germany and up to six in parts of southern Europe, but the number of these generations that are temporally coincident with *Bt*-maize MON810 pollen is unlikely to exceed two. Neglecting nonlinear effects, the mortality owing to *Bt*-maize MON810 might therefore represent just an additional 0.5 per cent per year. Again, neglecting density-dependent effects that might be important, a simplified analysis would predict that the expected population decline owing to maize MON810 would not exceed 5 per cent over 10 years. Such a small decline would be difficult to detect in practice (Aviron *et al*. 2009) because of the natural fluctuations and trends in lepidopteran populations (Conrad *et al*. 2006). Note that, by comparison, abiotic mortality factors analysed in field experiments with this species can reduce the larval population by more than 50 per cent (Annamalai *et al*. 1988) in one season. The impact of larval and pupal parasitoids can be even more effective in regulating *P. xylostella* population dynamics, since parasitization rates as high as 80 per cent are often found in field conditions (e.g. Talekar & Shelton 1983; Liu *et al*. 2000).

In principle, it should be possible to parametrize the model for other lepidopteran species, as long as there is sufficient information concerning mortality rates of individuals and concerning their host plants. Allowance may need to be made for the variability in susceptibility of lepidopteran larvae of a given species to *Bt*-maize pollen expressing Cry1Ab across Europe (Lozzia & Manachini 2003). The model provides a framework that can be used for future risk assessments concerning the exposure of non-target Lepidoptera to *Bt-*maize events that express different *Bt* proteins.

All modelling exercises are subject to uncertainties; as with any ecological model, further data would refine the estimates reported here. The variability in our reported estimates arises from: (i) natural variation between areas, reflecting expected agronomic and environmental heterogeneity, such as those relating to parameters *C*, *D*, *e*, *f*, etc.; (ii) differences between experts’ interpretation in parameters other than *g*(*E*) and *h*; and (iii) uncertainties arising from incomplete availability of data, particularly regarding sublethal effects.
